# Specificities and redundancies in the NEL family of bacterial E3 ubiquitin ligases of *Salmonella enterica* serovar Typhimurium

**DOI:** 10.3389/fimmu.2024.1328707

**Published:** 2024-02-01

**Authors:** Andrea Bullones-Bolaños, Paula Martín-Muñoz, Claudia Vallejo-Grijalba, Joaquín Bernal-Bayard, Francisco Ramos-Morales

**Affiliations:** Departamento de Genética, Facultad de Biología, Universidad de Sevilla, Sevilla, Spain

**Keywords:** *Salmonella enterica*, type III secretion systems, E3 ubiquitin ligase, SlrP, SspH1, SspH2

## Abstract

*Salmonella enterica* serovar Typhimurium expresses two type III secretion systems, T3SS1 and T3SS2, which are encoded in *Salmonella* pathogenicity island 1 (SPI1) and SPI2, respectively. These are essential virulent factors that secrete more than 40 effectors that are translocated into host animal cells. This study focuses on three of these effectors, SlrP, SspH1, and SspH2, which are members of the NEL family of E3 ubiquitin ligases. We compared their expression, regulation, and translocation patterns, their role in cell invasion and intracellular proliferation, their ability to interact and ubiquitinate specific host partners, and their effect on cytokine secretion. We found that transcription of the three genes encoding these effectors depends on the virulence regulator PhoP. Although the three effectors have the potential to be secreted through T3SS1 and T3SS2, the secretion of SspH1 and SspH2 is largely restricted to T3SS2 due to their expression pattern. We detected a role for these effectors in proliferation inside fibroblasts that is masked by redundancy. The generation of chimeric proteins allowed us to demonstrate that the N-terminal part of these proteins, containing the leucine-rich repeat motifs, confers specificity towards ubiquitination targets. Furthermore, the polyubiquitination patterns generated were different for each effector, with Lys48 linkages being predominant for SspH1 and SspH2. Finally, our experiments support an anti-inflammatory role for SspH1 and SspH2.

## 1 Introduction

*Salmonella enterica* serovar Typhimurium is an important bacterial pathogen that is responsible for millions of gastrointestinal infections every year in humans (1). As many other Gram negative pathogens and symbionts, *Salmonella* relies on type III secretion systems (T3SS) for its interactions with mammalian host cells (2). These systems, also known as injectisomes, are complex molecular devices (3) that allow some bacteria to inject specific proteins, known as effectors (4), directly into host cells. *S. enterica* possesses two distinct T3SS, T3SS1 and T3SS2, which are encoded by *Salmonella* pathogenicity island 1 (SPI1) and *Salmonella* pathogenicity island 2 (SPI2), respectively. T3SS1 is involved in host cell invasion and in initial colonization of the intestine (5). Some of the effectors secreted through this system are responsible for cytoskeletal rearrangements and the formation of membrane ruffles, which facilitate the entry of bacteria into host cells. T3SS2 is activated when *Salmonella* is inside the host cell and plays a crucial role in intracellular survival and replication of bacteria. The effectors of this system are involved in the generation of a specialized intracellular compartment called the *Salmonella*-containing vacuole (SCV), which is important for the evasion of host immune defenses (6).

Ubiquitination is a post-translational modification of proteins that involves the covalent attachment of one or more ubiquitins, small proteins consisting of 76 amino acids. It plays a pivotal role in the regulation of cellular processes such as protein degradation, signal transduction, and immune responses (7). Ubiquitin is bound to a protein in a sequential manner involving a ubiquitin-activating enzyme (E1), a ubiquitin-conjugating enzyme (E2), and a ubiquitin ligase (E3), which is essential for the selection of the specific target (8). Ubiquitin can be attached to target proteins as single ubiquitin molecules or as polyubiquitin chains. These chains can have different linkage types, with different roles (9). For example, Lys48 (K48)-linked ubiquitin chains are generally associated with protein degradation by the proteasome (10), and K63-linked chains are often involved in signaling processes (11). Although ubiquitination is commonly associated with eukaryotic cells, bacterial pathogens have evolved a variety of virulence factors, including T3SS effectors, to manipulate the host ubiquitination system (12, 13). Among them there are effectors with E3 ubiquitin ligase activity that are structurally similar to RING-type eukaryotic E3 ligases, such as NleG from enterohemorrhagic *Escherichia coli* (14), or HECT-type E3 ligases, such as SopA in *S. enterica* serovar Typhimurium (15). Interestingly, some effectors belong to a new family of E3 ligases that is not found in eukaryotic cells and is known as the NEL (for novel E3 ligase) family. Members of this family are the IpaH proteins from *Shigella flexneri*, as well as SlrP, SspH1, and SspH2 from *S. enterica* serovar Typhimurium (16).

In spite of their functional relationship with T3SS1 and T3SS2, the genes encoding these three effectors are located outside of SPI1 and SPI2, in other horizontally acquired regions of the chromosome (16). SlrP is secreted through T3SS1 and T3SS2 (17, 18), interacts with mammalian thioredoxin and catalyzes its ubiquitination, reducing its activity and leading to host cell death (19). This effector also binds to ERdj3, a chaperone located in the endoplasmic reticulum, and interferes with its folding activity, which can also contribute to cell death (20). A recently described ubiquitination target for SlrP is SNRPD2, a core component of the spliceosome (21). SspH1 is also secreted through both systems but localizes in the nucleus of host cells where it ubiquitinates PKN1 (22), leading to its proteasome-dependent degradation. SspH2 is specifically secreted through T3SS2 and interacts with the actin binding proteins filamin and profilin (23), the cell cycle regulator and NLR cochaperone SGT1 (24), and several other proteins (25). SspH2 and SGT1 form a trimeric complex with Nod1 leading to monoubiquitination and activation of the latter (24). Nod1 is a canonical NLR involved in the expression of pro-inflammatory chemokines such as IL-8 (26).

The presence of several effectors of one family in the same bacteria, displaying the same biochemical activity, as is the case for SlrP, SspH1, and SspH2 in *S. enterica* serovar Typhimurium, raises the interesting question of the degree of redundancy and specificity of these effectors. Here, we address this issue through a direct comparison of the three proteins in terms of patterns of expression, regulation, and translocation into eukaryotic cells. We also analyze their role in invasion and intracellular proliferation, and their ability to interact and ubiquitinate specific targets. Finally, we compare the ability of these effectors to interfere with the immune response of host cells.

## 2 Materials and methods

### 2.1 Bacterial strains and plasmids

The bacterial strains and plasmids used in this study are described in **Table 1**. *S. enterica* serovar Typhimurium strains derived from the wild-type strain 14028. Transductional crosses using the P22 HT105/1 *int-201* phage (37) were used for the construction of *Salmonella* strains.

**Table 1:** Bacterial strains and plasmids used in this study.

Strain/Plasmid	Relevant characteristics	Source/Reference
***Escherichia coli***		
BL21(DE3)	F^-^ *ompT gal dcm lon hsdS_B_* (r^-^ m^-^; *E. coli* B strain), with DE3, a λ prophage carrying the T7 RNA *pol* gene	Stratagene
DH5α	*supE44 ΔlacU*169 (Ø80 *lacZ*ΔM15) *hsdR17 recA1 endA1 gyrA96 thi-1 relA1*	(27)
ER2566	*fhuA2 lacZ::T7 gene1 [lon] ompT gal sulA11 R(mcr-73::miniTn10–TetS)2 [dcm] R(zgb-210::Tn10–TetS) endA1 Δ(mcrC-mrr)114::IS10*	New England Biolabs
XL1-Blue	*recA1 endA1 gyrA96 thi-1 hsdR17 supE44 relA1 Δlac-pro/*F’ *proAB lacI_q_ lacZ*ΔM15 Tn*10* (Tet^r^)	(28)
***Salmonella enterica*** **serovar Typhimurium**^**a**^		
14028	Wild type	ATCC
SV4676	*trg*:: Mu*d*J	(29)
SV5030	*slrP*::Cm^r^	Laboratory stock
SV5136	*ssaV*::Cm^r^	Laboratory stock
SV5193	*slrP*::3xFLAG Km^r^	(19)
SV5379	*prgH*	Laboratory stock
SV5452	*ssrB*::Cm^r^	(30)
SV5604	*prgH ssaV*::Cm^r^	Laboratory stock
SV6016	*slrP*::mini-Tn*5cyaA’*	(31)
SV9213	*phoP*::Km^r^	Laboratory stock
SV9988	*sspH2*::Km^r^	This work
SV9998	*sspH1*::Km ^r^	This work
SV10107	*sspH1*::3xFLAG Km ^r^	This work
SV10108	*sspH2*::3xFLAG Km ^r^	This work
SV10109	*slrP*::Cm ^r^ *sspH1*::Km ^r^	This work
SV10110	*slrP*::Cm ^r^ *sspH2*::Km ^r^	This work
SV10114	*sspH1 sspH2*::Km ^r^	This work
SV10117	*slrP sspH1 sspH2*::Km ^r^	This work
SV10239	*sspH1*::CyaA’	This work
SV10240	*sspH2*::CyaA’	This work
***Plasmids***		
pCS2+	Mammalian expression vector	F. Romero
pGAD1318	Yeast two-hybrid vector, Ap ^r^	(32)
pGEX-4T-1	GST fusion vector, Ap ^r^	GE Healthcare
pIZ1628	pLEX10-SlrP	(21)
pIZ1673	pSIF003-R1 Δ*lacI*	(33)
pIZ1749	pQE30-SlrP	(21)
pIZ1917	pIZ1673-SlrP (1–180)	Laboratory stock
pIZ2149	pSB377-P*slrP*	This work
pIZ2370	pGAD1318-SNRPD2	(21)
pIZ3403	pGEX-4T-2-SNRPD2	(21)
pIZ3407	pLEX10-SspH1	(21)
pIZ3408	pLEX10-SspH2	(21)
pIZ3597	pQE80L-SspH1	(21)
pIZ3598	pQE80L-SspH1	(21)
pIZ3639	pSB377-P*sspH2*	This work
pIZ3640	pIZ1673-SspH1 (1–197)	This work
pIZ3641	pIZ1673-SspH2 (1–203)	This work
pIZ3643	pLEX10-SlrP (457–765)	This work
pIZ3644	pLEX10-SlrP (1–456)	This work
pIZ3645	pLEX10-SspH1 (399–700)	This work
pIZ3646	pLEX10-SspH2 (486–788)	This work
pIZ3649	pSB377-P*sspH1*	This work
pIZ3657	pCS2-HA-UBC	This work
pIZ3663	pLEX10-SlrP (1–456)/SspH1 (399–700)	This work
pIZ3668	pLEX10-SspH1 (1–398)	This work
pIZ3669	pQE80L-SlrP (1–456)/SspH2(486-788)	This work
pIZ3670	pQE80L-SspH1(1-398)/SlrP(457-765)	This work
pIZ3671	pQE80L-SspH1(1-398)/SspH2(486-788)	This work
pIZ3672	pLEX10-SspH2(1-485)	This work
pIZ3675	pLEX10-SspH1(1-398)-SlrP(457-765)	This work
pIZ3681	pLEX10-SspH2(1-485)/SspH1(399-700)	This work
pIZ3682	pCS2-HA-UBC(K48R)	This work
pIZ3683	pCS2-HA-UBC(K63R)	This work
pIZ3686	pLEX10-SspH2(1-485)/SlrP(457-765)	This work
pIZ3687	pQE80L-SspH2(1-485)/SlrP(457-765)	This work
pIZ3692	pQE80L-SlrP(1-456)/SspH1(399-700)	This work
pIZ3693	pQE80L-SspH2(1-485)/SspH1(399-700)	This work
pIZ3695	pTYB21-HA-UBC(K48R)	This work
pIZ3696	pTYB21-HA-UBC(K63R)	This work
pIZ3698	pTYB21-HA-UBC	This work
pIZ3703	pLEX10-SlrP(1-456)-SspH2(486-788)	This work
pIZ3706	pLEX10-SspH1(1-398)/SspH2(486-788)	This work
pIZ3712	pGEX-4T-1/PKN1(HR1b)	This work
pIZ3716	pTYB21-HA-UBC(K48R/K63R)	This work
pLEX10	Yeast two-hybrid vector, Ap ^r^	(34)
pQE80L	6His fusion vector, Ap ^r^	Qiagen
pSB377	Parent for *luxCDABE* transcriptional fusions, Ap ^r^	(35)
pSIF003-R1	pEX-CyaA (1–412) derivative	(36)
pTYB21	Vector for generation of intein fusions	New England Biolabs

a Derivatives of these strains were used as indicated in the text.

### 2.2 Bacterial culture

The standard culture medium for bacteria was LB broth. For SPI1-inducing conditions, *S. enterica* strains were grown overnight at 37°C in LB-0.3 M NaCl medium without shaking. For SPI2-inducing conditions, the bacteria were inoculated in low magnesium minimal medium (LPM) at pH 5.8, and incubated at 37°C with shaking. LPM contained 80 mM 2-(N-morpholino) ethanesulfonic acid (pH 5.8), 5 mM KCl, 7.5 mM (NH_4_)_2_SO_4_, 0.5mM K_2_SO_4_, 0.1% casamino acids, 38 mM glycerol, 337.5 μM K_2_HPO_4_-KH_2_PO_4_ (pH 7.4), and 8 μM MgCl_2_. The solid media contained 1.5% agar. Antibiotics were used at the following concentrations in LB: kanamycin (Km), 50 μg/ml; chloramphenicol (Cm), 20 μg/ml; ampicillin (Ap), 100 μg/ml. In minimal medium antibiotics were used at these concentrations: Km, 125 μg/ml; Cm, 5 μg/ml; Ap, 50 μg/ml.

### 2.3 Yeast strains, culture, and two-hybrid methods

The *Saccharomyces cerevisiae* strain used was L40 (38). The culture media for yeasts were YPD: 1% yeast extract, 2% peptone, 2% glucose; and synthetic drop-out (SD) medium: 0.15% yeast nitrogen base without amino acids and ammonium sulfate, 0.5% ammonium sulfate, 2% glucose; and yeast synthetic drop-out supplements (Formedium) lacking the appropriate components: tryptophan to select for the presence of derivatives of pLEX10 (34), leucine for derivatives of pGAD1318 (32), and histidine to check interactions. Solid medium contained 1.5% agar. For two-hybrid assays, strain L40 was transformed with the appropriate combinations of pLEX10 and pGAD1318 derivatives using the lithium acetate procedure (39). The transformants were selected in SD without tryptophan and leucine. Interactions were analyzed by checking the growth in SD lacking tryptophan, leucine, and histidine.

### 2.4 Mammalian cell culture, lysis, transfection, and analysis of cytokine secretion

HeLa (human epithelial; ECACC no. 93021013), RAW264.7 (murine macrophages; ECACC no. 91062702), and NRK-49F (normal rat kidney fibroblasts; ATCC CRL-1570) cells were cultured in DMEM supplemented with 10% fetal calf serum, 2 mM L-glutamine, 100 U/ml penicillin, and 100 μg/ml streptomycin. The cells were kept in a humidified atmosphere with 5% CO_2_ at 37°C. For cell lysis, 2 × 10^7^ to 10^8^ cells per ml were incubated at 4°C in NP40 buffer (10 mM Tris-HCl pH 7.4, 150 mM NaCl, 10% glycerol, 1% NP40, 1 mM PMSF, 1% protease inhibitor cocktail P8849 from Sigma-Aldrich) for 20 min. The extract was centrifuged at 20,000× g for 20 min and the supernatant was stored at -80°C. For transient transfection assays, 2–5 × 10^6^ HeLa cells/assay were resuspended in 200 μL of 15 mM HEPES-buffered serum-containing medium, mixed with 50 μL of 210 mM NaCl containing 5–10 μg of plasmid DNA and electroporated using a BTX Electrocell Manipulator 600 set at 240 V, 950 μF, resistance = None. Cells were processed 24 h after electroporation. The supernatants of transfected cells were tested for CCL5 secretion using the human CCL5/RANTES DuoSet ELISA kit (R&D Systems) and for the secretion of IFNγ, IL-1β, IL-6 and IL-8 using the appropriate uncoated ELISA kits (Thermo Fisher Scientific).

### 2.5 Bacterial infection of cultured cells, invasion, proliferation, and protein translocation assays

Mammalian cells were plated in 24-well plates at 1.5 x 10^5^ cells per well and incubated 24 h at 37°C with 5% CO_2_ in media without antibiotics. For infections under SPI1-inducing (invasive) conditions, bacteria grown overnight in LB with 0.3 M NaCl in a tightly closed tube without shaking were added at a multiplicity of infection of 150. For infections of RAW264.7 cells under non-invasive conditions, the bacteria were grown in LB for 24 h at 37°C with shaking. Cell culture was washed twice with phosphate-buffered saline (PBS) at 1 h post-infection (p.i.), overlaid with DMEM containing 100 μg/ml gentamicin, and incubated for another hour. The culture was then washed twice with PBS, covered with DMEM with gentamicin 16 μg/ml, and incubated for 6 h. Following the infections described above, the translocation of CyaA’ fusions into eukaryotic cells was monitored by measuring the levels of cyclic AMP (cAMP) 2 h or 8 h p.i. Infected cells were lysed and the level of cAMP in the lysates was determined using a colorimetric direct cAMP enzyme immunoassay kit (Arbor Assays) according to the manufacturer’s instructions. For invasion and proliferation assays, infections were carried out using a 10:1 mix of a mutant strain and a *trg*::Mu*d*J mutant (wild type for invasion and intracellular proliferation, but Lac^+^ due to the Mu*d*J insertion). Competitive indices for invasion and proliferation were calculated as previously described (29) after plating appropriate dilutions and enumerating white colonies and blue colonies (*trg*::Mu*d*J) in LB plates supplemented with 40 μg/ml 5-bromo-4-chloro-galactopyranoside (X-Gal). For invasion, the input was the initial mix of bacteria used in the infection and the output, bacteria recovered 2 h p.i. For intracellular proliferation, bacteria were recovered 2 h p.i. (input) and 24 h p.i. (output).

### 2.6 Luminescence measurements

*Salmonella* strains were grown in triplicate in the appropriate media and samples of 150 μl of each culture were used to measure luminescence and OD_600_, in 96-well white clear bottom plates using a FLUOstar Omega plate reader (BMG LABTECH). To measure the luminescence of intracellular bacteria, RAW264.7 macrophages were plated in 96-well white clear bottom plates at 3 x 10^4^ cells per well, and were infected 24 h later with non-invasive bacteria (grown in LB for 24 h at 37°C with shaking) at a multiplicity of infection of 500. The cell culture was washed twice with PBS 30 min p.i., overlaid with DMEM containing 100 μg/ml gentamicin, and incubated for 1.5 h. The culture was then washed twice with PBS, covered with DMEM with 16 μg/ml gentamicin and incubated for 6 additional h. Luminescence was measured at 2, 4 and 8 h p.i. and the colony forming units per well were calculated after incubation with 1% Triton X-100 in PBS for 10 min at 37°C to release bacteria, plating appropriate dilutions in LB with Ap, and counting colonies after 24 h incubation at 37°C.

### 2.7 DNA amplification with the polymerase chain reaction, cloning, mutagenesis, and sequencing

Amplification reactions were carried out on a T100 Thermal Cycler (Bio-Rad) using Q5 High-Fidelity DNA polymerase (New England Biolabs) or MyTaq Red DNA polymerase (Bioline) according to the supplier’s instructions. Oligonucleotides are described in **Table 2**. Plasmid construction was carried out either using a classical cloning strategy based on enzymatic digestion or by Gibson assembly (40). The plasmids were sequenced with an automated DNA sequencer (Stab Vida). The disruption of *sspH1* or *sspH2* and replacement in strain 14028 with a gene conferring resistance to Km was carried out as previously described (41) using primers listed in **Table 2** and plasmid pKD4. The antibiotic resistance cassette introduced by the gene targeting procedure was eliminated by recombination using the FLP helper plasmid pCP20. The addition of a DNA fragment encoding the 3xFLAG epitope tag at the 3’ end of *sspH1* or *sspH2* was carried out as described (42) using primers listed in **Table 2** and the plasmid pSUB11. The protocol to generate chromosomal *cyaA’* translational fusions was also previously described (43). Overlapping PCR was used to generate point mutations in the gene encoding human ubiquitin, *UBC*, using pIZ3683 as template and primers indicated in **Table 2**, and the PCR products were cloned in pCS2 with EcoRI/XbaI restriction endonucleases. The constructs for the purification of HA ubiquitin were made by Gibson assembly using primers P2pTYB21-Fw and P1pTYB21-Rv to amplify the plasmid pTYB21, and primers P1pTYB21-HA-UB-Fw and P2pTYB21-HA-UB-Rv to amplify the different ubiquitins of plasmids pIZ3657, pIZ3682, and pIZ3683. Double mutant ubiquitin was constructed by overlapping PCR using pIZ3683 as a template with the following primers: PCR1: P1-pTYB21-HA-UB-Fw and UB-K48R-Rv; PCR2: UB-K48R-Fw and pCS2-3; PCR3: P1-pTYB21-HA-UB-Fw and P1-pTYB21-HA-UB-Rv and cloning into pTYB21 by Gibson assembly. The presence of the inserts was confirmed with primers pTYB21-ext-Fw and pTYB21-ext-Rv.

**Table 2:** Oligonucleotides used in this study.

Oligonucleotide / use	Sequence 5’-3’
*sspH1 deletion*
sspH1H1P1	TTAATCTCTTTTCATTGTGCTGTAAATTAGGCAGTGGAATGTGTAGGCTGGAGCTGCTTC
sspH1H2P2	TTCACCGCACCACATTCGCCTGGTGCGGTGAATATCGTGCCATATGAATATCCTCCTTAG
*sspH2 deletion*	
sspH2H1P1	CGGACAGATACTATATGTAAATTTATAAAGGTTTTTTGTTGTGTAGGCTGGAGCTGCTTC
sspH2H2P2	GGAATATCTTTGTCGCACCGCACCTCATTCACCTGGTGCACATATGAATATCCTCCTTAG
*Epitope tagging of SspH1*	
sspH1-3xFLAG-5’	CTGGGTAGCTATCTGACAGCCCGGTGGCGTCTTAACGACTACAAAGACCATGACGG
sspH1-3xFLAG-3’	CCGCACCACATTCGCCTGGTGCGGTGAATATCGTGCCATATGAATATCCTCCTTAG
*Epitope tagging of SspH2*	
sspH2-3xFLAG-5’	CTGGGGAGCTATCTGAACGTTCAGTGGCGTCGTAACGACTACAAAGACCATGACGG
sspH2-3xFLAG-3’	TATCTTTGTCGCACCGCACCTCATTCACCTGGTGCACATATGAATATCCTCCTTAG
*Chromosomal sspH1::cyaA’ fusion*	
ssph1-197cyaP1	AGTGGTACAGGAAATGCGTGATTGCCTGAATAACGGCAATCTGCAGCAATCGCATCAGGC
ssph1-197cyaP2	GTAAGGTGGTAAGACCTGACGCTCCCACGTTAAGCACTGGTTAGAAAAACTCATCGAGCATC
*Chromosomal sspH2::cyaA’ fusion*	
ssph2-203cyaP1	AGTGGTACAGAAAATGCGTGCCTGCCTGAATAATGGCAATCTGCAGCAATCGCATCAGGC
ssph2-203cyaP2	GCAAGGTGGTAAGACCTGATTCTCCCACGTTAAGCACTGCTTAGAAAAACTCATCGAGCATC
*Construction of pIZ2149*	
PslrPfwEco	CATGGAATTCCGATCGCCAGCGAGTCATCG
PslrPrevEco	GATCGAATTCATTTTCCCTACCTGATCTG
*Construction of pIZ3639*	
psspH2Ecofw	ACGTGAATTCAAAGGGTTTATTCGCCGGAAG
psspH2Ecorv	AGCTGAATTCAACAAAAAACCTTTATAAATTTACATATAG
*Construction of pIZ3640*	
ssph1SDbamh1fw	GAATGGATCCAGGAGGTGGAATATGTTTAATATCCGC
ssph1-197bamh1rv	CAAGGGATCCGATTGCCGTTATTCAGGCAATC
*Construction of pIZ3641*	
ssph2SDbamh1fw	GAATGGATCCAGGAGGTTTGTTATGCCCTTTCATATTGG
ssph2-203bamh1rv	CAAGGGATCCCATTGCCATTATTCAGGCAGGC
*Construction of pIZ3643*	
slrP457bamHIfw	AGTCGGATCCTCAATTGTTCGGGTAACTCG
slrPsalIrv	AGTCGTCGACCTATCGCCAGTAGGCGCTCATG
*Construction of pIZ3644*	
slrPecoRIfw	AGTCGAATTCATGTTTAATATTACTAATATACAATC
slrP456bamHIrv	AGTCGGATCCAAAGTCGCCCATGGCAAACAATAC
*Construction of pIZ3645*	
sspH1-399bamHIfw	AGTCGGATCCTCCGTCCCCCGGGAAGCCCG
sspH1xhorv	TGACCTCGAGTCAGTTAAGACGCCACCGGG
*Construction of pIZ3646*	
sspH2-486bamHIfw	AGTCGGATCCGCCTCCGCCCCCCGGGAAAC
sspH2salrv	GATCGTCGACTCAGTTACGACGCCACTGAAC
*Construction of pIZ3649*	
PsspH1EcoFw	ATGCGAATTCAGCGCTGTTTTGCCTGGCTG
psspH1Ecorv	ATGCGAATTCATTCCACTGCCTAATTTACAG
*Construction of pIZ3657*	
BamHI-kozak-HA-Ub-Fw	ACGTGGATCCGCCGCCACCATGTACCCTTATGATGTACCAGACTACGCTGGCCGG
ubqXbaIrv	GATCTCTAGATCACCCACCTCTGAGACGGAGCAC
*Construction of pIZ3663*	
slrPecoRIfw	AGTCGAATTCATGTTTAATATTACTAATATACAATC
slrP456bamHIrv	AGTCGGATCCAAAGTCGCCCATGGCAAACAATAC
sspH1-399bamHIfw	AGTCGGATCCTCCGTCCCCCGGGAAGCCCG
sspH1xhorv	TGACCTCGAGTCAGTTAAGACGCCACCGGG
*Construction of pIZ3668*	
sspH1bamfw	ATGCGGATCCATGTTTAATATCCGCAATAC
sspH1-398bamHIrv	AGTCGGATCCAGGCCCCGCCATATCGAAGTG
*Construction of pIZ3669*	
SlrPBamG5	CTGAGGATCCATGTTTAATATTACTAATATACAATC
slrp456sacIsalIrv	AGTCGTCGACGAGCTCAAAGTCGCCCATGGCAAACAATAC
sspH2-486salIfw	AGTCGTCGACGCCTCCGCCCCCCGGGAAAC
sspH2hindIIIrv	AGTCAAGCTTTCAGTTACGACGCCACTGAAC
*Construction of pIZ3670*	
sspH1bamfw	ATGCGGATCCATGTTTAATATCCGCAATAC
sspH1-398salIsacIrv	AGTCGAGCTCGTCGACAGGCCCCGCCATATCGAAGTG
slrP457salIfw	AGTCGTCGACTCAATTGTTCGGGTAACTCG
slrPhindIIIrv	AGTCAAGCTTCTATCGCCAGTAGGCGCTCATG
*Construction of pIZ3671*	
sspH1bamfw	ATGCGGATCCATGTTTAATATCCGCAATAC
sspH1-398salIsacIrv	AGTCGAGCTCGTCGACAGGCCCCGCCATATCGAAGTG
sspH2-486salIfw	AGTCGTCGACGCCTCCGCCCCCCGGGAAAC
sspH2hindIIIrv	AGTCAAGCTTTCAGTTACGACGCCACTGAAC
*Construction of pIZ3672*	
sspH2ecofw	ATGCGAATTCATGCCCTTTCATATTGGAAG
sspH2-485bamHIrv	AGTCGGATCCTCCCGCCATATCGAATCGTATTATG
*Construction of pIZ3675*	
sspH1bamfw	ATGCGGATCCATGTTTAATATCCGCAATAC
sspH1-398bamHIrv	AGTCGGATCCAGGCCCCGCCATATCGAAGTG
slrP457bamHIfw	AGTCGGATCCTCAATTGTTCGGGTAACTCG
slrPsalIrv	AGTCGTCGACCTATCGCCAGTAGGCGCTCATG
*Construction of pIZ3681*	
sspH2ecofw	ATGCGAATTCATGCCCTTTCATATTGGAAG
sspH2-485bamHIrv	AGTCGGATCCTCCCGCCATATCGAATCGTATTATG
sspH1-399bamHIfw	AGTCGGATCCTCCGTCCCCCGGGAAGCCCG
sspH1xhorv	TGACCTCGAGTCAGTTAAGACGCCACCGGG
*Construction of pIZ3682*	
UbqEcoFw	GATCGAATTCATGCAGATCTTCGTGAAGACC
UB-K48R-Rv	CCATCTTCCAGCTGTCGCCCAGCAAAGATCAAC
Ub-K48R-Fw	GTTGATCTTTGCTGGGCGACAGCTGGAAGATGG
pCS2-3	TGACCATGATTACGCCAAGC
*Construction of pIZ3683*	
UbqEcoFw	GATCGAATTCATGCAGATCTTCGTGAAGACC
Ub-K63R-Rv	GCAGGGTGGACTCTCGCTGGATGTTGTAGTC
Ub-K63R-Fw	GACTACAACATCCAGCGAGAGTCCACCCTGC
pCS2-3	TGACCATGATTACGCCAAGC
*Construction of pIZ3686*	
sspH2ecofw	ATGCGAATTCATGCCCTTTCATATTGGAAG
sspH2-485bamHIrv	AGTCGGATCCTCCCGCCATATCGAATCGTATTATG
slrP457bamHIfw	AGTCGGATCCTCAATTGTTCGGGTAACTCG
slrPsalIrv	AGTCGTCGACCTATCGCCAGTAGGCGCTCATG
*Construction of pIZ3687*	
sspH2Bamfw	ATGCGGATCCATGCCCTTTCATATTGGAAG
sspH2-485sacIsalIrv	AGTCGTCGACGAGCTCTCCCGCCATATCGAATCGTATTATG
slrP457salIfw	AGTCGTCGACTCAATTGTTCGGGTAACTCG
slrPhindIIIrv	AGTCAAGCTTCTATCGCCAGTAGGCGCTCATG
*Construction of pIZ3692*	
nSlrP-SacI-Fw	GTCAGAGCTCATGTTTAATATTACTAATATACAATCTACG
sspH1hindIIIrv	AGTCAAGCTTTCAGTTAAGACGCCACCGGG
*Construction of pIZ3693*	
sspH2Bamfw	ATGCGGATCCATGCCCTTTCATATTGGAAG
sspH2-485sacIsalIrv	AGTCGTCGACGAGCTCTCCCGCCATATCGAATCGTATTATG
sspH1-399sacIfw	AGTCGAGCTCTCCGTCCCCCGGGAAGCCCG
sspH1hindIIIrv	AGTCAAGCTTTCAGTTAAGACGCCACCGGG
*Construction of pIZ3695, pIZ3696, pIZ3698*	
P2-pTYB21-Fw	TAAATAACTAGTTGATCCGGCTGC
P1-pTYB21-Rv	GTTCTGTACAACAACCTGAGATCC
P1pTYB21-HA-UB-Fw	GTTGTTGTACAGAACATGTACCCTTATGATGTACCAGAC
P2pTYB21-HA-UB-Rv	TCAACTAGTTATTTACCCACCTCTGAGACGGAGC
*Construction of pIZ3703*	
slrPecoRIfw	AGTCGAATTCATGTTTAATATTACTAATATACAATC
slrP456bamHIrv	AGTCGGATCCAAAGTCGCCCATGGCAAACAATAC
sspH2-486bamHIfw	AGTCGGATCCGCCTCCGCCCCCCGGGAAAC
sspH2salrv	GATCGTCGACTCAGTTACGACGCCACTGAAC
*Construction of pIZ3706*	
sspH1bamfw	ATGCGGATCCATGTTTAATATCCGCAATAC
sspH1-398bamHIrv	AGTCGGATCCAGGCCCCGCCATATCGAAGTG
sspH2-486bamHIfw	AGTCGGATCCGCCTCCGCCCCCCGGGAAAC
sspH2salrv	GATCGTCGACTCAGTTACGACGCCACTGAAC
*Construction of pIZ3716*	
P1pTYB21-HA-UB-Fw	GTTGTTGTACAGAACATGTACCCTTATGATGTACCAGAC
UB-K48R-Rv	CCATCTTCCAGCTGTCGCCCAGCAAAGATCAAC
Ub-K48R-Fw	GTTGATCTTTGCTGGGCGACAGCTGGAAGATGG
pCS2-3	TGACCATGATTACGCCAAGC

### 2.8 Expression and purification of HA-ubiquitin

The plasmids pIZ3698, pIZ3695, pIZ3696, and pIZ3716 were transformed into *E. coli* ER2566. Overnight cultures of these strains were diluted 50 times in 500 ml of ampicillin-supplemented LB and incubated at 37°C until an OD_600_ of 0.5. The expression of target proteins was induced by adding 0.4 mM isopropyl-β-D-thiogalactoside and incubating overnight at 20°C with shaking. Subsequently, the cultures were centrifuged at 5000 g, 15 min, 4°C, the pellets were washed with PBS and centrifuged again to remove PBS. The pellets were lysed by sonication (output 2, duty cycle 30%, 1 min, 5 times) in column buffer (10 mM Tris-HCl pH 7.5, 150 mM NaCl, 10% glycerol, 0.5% NP40) containing 1 mM PMSF and protease inhibitor cocktail (1:200) (Sigma-Aldrich). The supernatants were separated from the pellets by centrifugation at 15000 g, 20 min, 4°C and loaded into the affinity chromatography column according to the IMPACT E6901 Manual (New England Biolabs), with the following modifications: crystal columns of 1.5 x 20 cm and 5 ml of chitin beads were used. To release target proteins from the column, beads were incubated with cleavage buffer with DTT 100 mM, 72 h at 4°C. The eluted fractions were dialyzed in 10 mM HEPES buffer pH 7.5 with 5% glycerol using the Slide-A-Lyzer^®^ Dialysis cassette 3,500 MWCO, 3-12 ml capacity and incubated overnight at 4°C with slight shaking. To concentrate the proteins centrifugal filter units (Amicon^®^ Ultra-15) were used. The aliquots were stored at -80°C and the protein concentration was quantified using the Coomassie (Bradford) protein assay kit from Thermo Scientific.

### 2.9 Purification of fusion proteins, electrophoresis, and immunoblotting

The expression of GST fusion proteins was induced by the addition of 1 mM isopropyl-β-D-thiogalactoside to *E. coli* BL21 (DE3) containing pGEX-4T-1, pGEX-4T-2 or their derivatives. Bacteria were sonicated in NP40 buffer and fusion proteins were isolated from bacterial lysates by affinity chromatography with glutathione-agarose beads (Sigma-Aldrich). Proteins tagged with 6His were produced after the addition of 1 mM isopropyl-β-D-thiogalactoside to *E. coli* XL1-Blue containing derivatives of pQE30 or pQE80L, purified with Ni-NTA agarose beads (Sigma-Aldrich) and eluted with 300 mM imidazole in binding buffer (50 mM NaH_2_PO_4_, 300 mM NaCl). For electrophoresis, bacterial and mammalian lysates were mixed with an equal volume of 4x Laemmli sample buffer and boiled for 5 min. Proteins were separated by SDS-PAGE using mini-protean TGX precast gels, 4-15% gradient (BioRad), and electrophoretically transferred to nitrocellulose filters for western blot analysis. Primary antibodies were: anti-FLAG M2 (mouse, monoclonal, 1:5,000, Sigma-Aldrich), anti-HA-peroxidase 3F10 (rat, 1:2000, Roche), anti-GroEL (rabbit, polyclonal, 1:20,000, Sigma-Aldrich), anti-DnaK 8E2/2 (mouse, monoclonal, 1:5,000, Assay Designs). Secondary antibodies were goat anti-mouse IRDye 800CW-conjugated or goat anti-rabbit IRDye 680RD-conjugated antibodies (LI-COR). The bands were detected using the Odyssey Fc imaging system (LI-COR).

### 2.10 *In vitro* ubiquitination assays

Ubiquitination reactions were carried out in a 20-μl mixture containing buffer A (25 mM Tris-HCl, pH 7.5, 50 mM NaCl, 5 mM ATP, 10 mM MgCl_2_, 0.1 mM DTT), 2 μg of HA-tagged ubiquitin, 0.25 μg of E1 (Boston Biochem, Cambridge, MA, USA) and 1 μg of E2 (human recombinant UbcH5b from Boston Biochem) in the presence or absence of ubiquitin ligases and substrates. Reactions were incubated at 37°C for 1 h with shaking and stopped by adding an equal volume of Laemmli sample buffer 4X containing 100 mM DTT and boiling. Some reactions were carried out with GST fusion proteins bound to glutathione-agarose beads, and the beads were washed five times with NP40 buffer before boiling in Laemmli sample buffer with 100 mM DTT.

### 2.11 Statistical analysis

Each competitive index and each cytokine concentration value are the mean of at least three independent infections or transfections. Means and standard deviations were calculated and a Student’s *t*-test was used to evaluate if each competitive index was significantly different from 1 or if every cytokine concentration was significantly different from the concentration in the control assay. *P* values of 0.05 or less are considered significant.

## 3 Results

### 3.1 *In vitro* expression and regulation of *slrP*, *sspH1*, and *sspH2*

**Figure 1:**
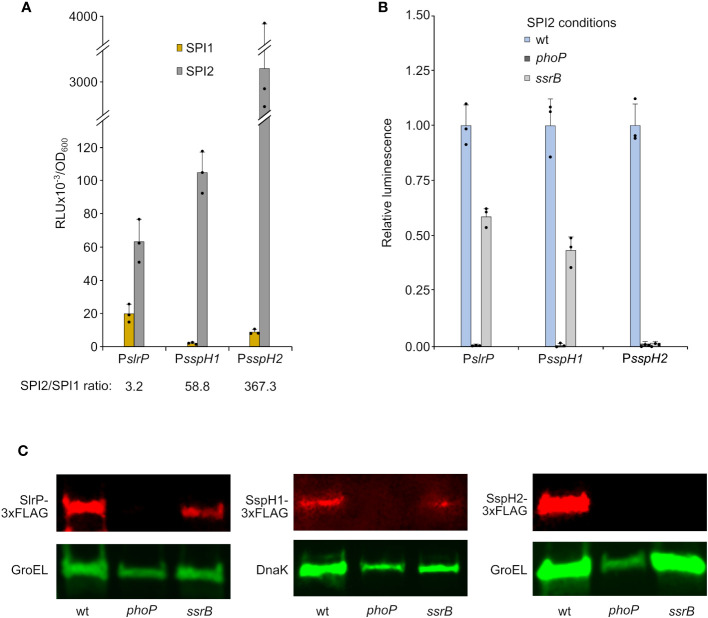
Expression and regulation of *slrP*, *sspH1*, and *sspH2*. DNA fragments containing the promoters of *slrP*, *sspH1*, or *sspH2* were cloned into plasmid pSB377 to generate *luxCDABE* transcriptional fusions. **(A)** Luminescence was measured in cultures of *S. enterica* serovar Typhimurium strain 14028 carrying these plasmids grown to stationary phase in LB 0.3 M NaCl (SPI1-inducing conditions) and LPM (SPI2-inducing conditions). **(B)** Luminescence was measured under SPI2-inducing conditions in different genetic backgrounds: wild-type (wt), *phoP* null mutant or *ssrB* null mutant. RLU, relative light units. Means and standard deviations of three independent measurements are represented. Dots represent individual values. **(C)** Protein extracts of derivatives of *S. enterica* serovar Typhimurium strains producing 3xFLAG-tagged SlrP, SspH1, or SspH2 grown under SPI2-inducing conditions were resolved by SDS-PAGE. Immunoblotting was performed with monoclonal anti-FLAG antibodies. Anti-GroEL or anti-DNAK antibodies were used as loading control. Replicate experiments for this panel are shown in **Supplementary Material**.

*S. enterica* serovar Typhimurium has three orthologous genes, *slrP*, *sspH1*, and *sspH2*, which encode E3 ubiquitin ligases belonging to the NEL family of type III secretion effectors. To compare the expression of these genes, we cloned their promoter regions into the plasmid pSB377 to generate *luxCDABE* transcriptional fusions. Plasmids were introduced into *S. enterica* serovar Typhimurium strain 14028 and luminescence was measured after growth under conditions inducing SPI1 expression (LB with 0.3 M NaCl without aeration) and conditions inducing SPI2 expression (LPM at pH 5.8 with high aeration). Although the three genes were preferentially expressed under SPI2-inducing conditions (**Figure 1A**), there were dramatic differences in the ratios between SPI2- and SPI1-inducing conditions. While *slrP* was significantly expressed under SPI1-inducing conditions and the SPI2/SPI1 ratio was only 3.2, expression of *sspH1* and *sspH2* was more restricted and the SPI2/SPI1 ratios were 58.8 and 367.3, respectively. Luminescence was also measured using *Salmonella* strains lacking virulence regulators PhoP or SsrB. This analysis revealed that the two-component system PhoQ/PhoP was strictly necessary for the expression of *slrP*, *sspH1*, and *sspH2* under SPI2-inducing conditions. Furthermore, SsrB, the positive regulator of SPI2, was essential for the expression of *sspH2*, but has only a partial influence on the expression of *slrP* and *sspH1* (**Figure 1B**). These results were confirmed at the protein level by immunoblot using 3xFLAG chromosomal fusions (**Figure 1C**).

### 3.2 Expression and regulation of *slrP*, *sspH1*, and *sspH2* in host cells

**Figure 2:**
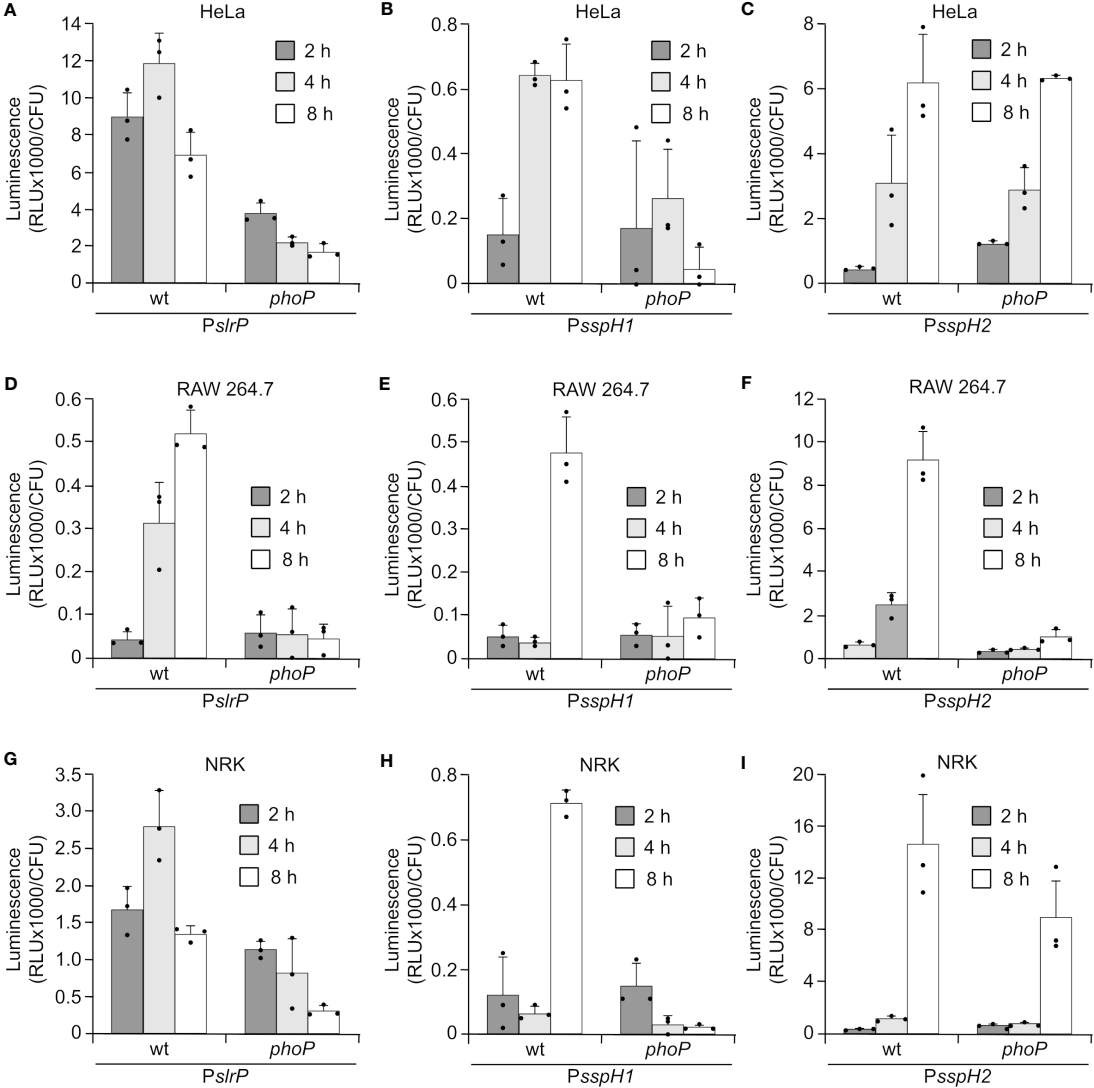
Expression of *slrP*, *sspH1*, and *sspH2* during host cells infection. *S. enterica* serovar Typhimurium carrying plasmids expressing P*slrP::luxCDABE* **(A, D, G)**, P*sspH1:luxCDABE* **(B, E, H)** or P*sspH2::luxCDABE* **(C, F, I)** were grown under invasive conditions (16 h in LB with 0.3 M NaCl without aeration) to infect HeLa cells **(A–C)** and NRK cells **(G–I)**, or non-invasive conditions (24 h in LB at 37°C with aeration) to infect RAW264.7 macrophages **(D–F)**. Luminescence produced by intracellular bacteria was measured 2, 4, and 8 h p.i.. Dots represent individual values.

*Salmonella* carrying transcriptional *lux* fusions were also used to infect cultures of epithelial HeLa cells, NRK fibroblasts, and RAW264.7 macrophages. Luminescence measurements at different times p.i. suggested that in most cases maximal expression was reached after 8 h (**Figure 2**), when *Salmonella* inside the SCV is also expressing SPI2. *In vivo* expression inside macrophages required activation by PhoP, which is consistent with the *in vitro* expression and regulation data shown in the previous section. However, expression of *sspH2* inside HeLa and NRK cells was at least partially independent of PhoP.

### 3.3 Patterns of translocation of SlrP, SspH1, and SspH2 into host cells

**Figure 3:**
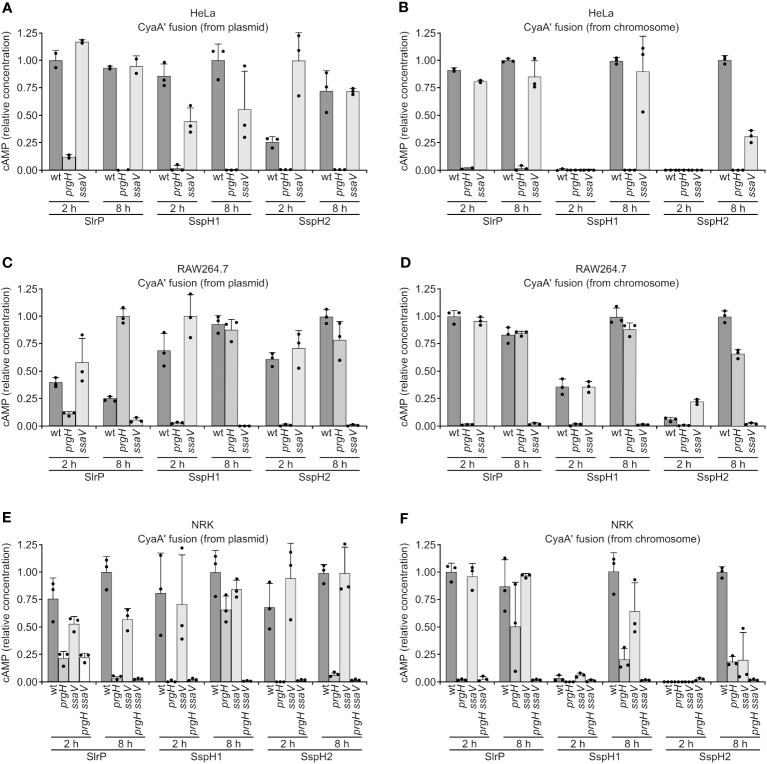
Translocation of SlrP, SspH1 and SspH2 into mammalian cells. Human epithelial HeLa cells **(A, B)**, RAW264.7 murine macrophages **(C, D)**, and NRK rat kidney fibroblasts **(E, F)** were infected with derivatives of *S. enterica* serovar Typhimurium 14028 (wild-type, wt*, prgH*, *ssaV*, and *prgH ssaV* strains) carrying a plasmid expressing SlrP-CyaA’, SspH1-CyaA’, or SspH2-CyaA’ fusions from a constitutive promoter **(A, C, E)** or chromosomal fusions expressed under native promoters **(B, D, F)**. Bacteria were grown under SPI1-inducing conditions (invasive conditions) except for infections of RAW264.7 cells for 8 h (non-invasive conditions to prevent early cell death). Levels of cAMP were measured as and indication of translocation. For each effector and cell type, these levels were relativized to the level obtained for the wild-type infection at 2 h or 8 h, that was set to 1. Means and standard deviations from triplicate experiments are represented. Dots represent individual values.

The translocation of the three effectors into host cells was studied using two types of CyaA’ translational fusions: plasmid fusions under the control of a constitutive promoter, and chromosomal fusions controlled by native promoters. Fusions were introduced into wild-type *Salmonella*, as well as a *prgH* mutant (lacking T3SS1) and a *ssaV* mutant (lacking T3SS2), and these bacteria were used to infect human epithelial HeLa cells, murine RAW264.7 macrophages, and rat NRK fibroblasts. Translocation was detected by an increase in cAMP concentration in cell cultures that was measured 2 h and 8 h p.i. The bacteria were grown under SPI1-inducing conditions (invasive conditions) except for RAW264.7 8 h infections to avoid triggering early pyroptosis. As seen in **Figure 3**, the observed translocation patterns were very complex. Some general conclusions are: (i) translocation is more promiscuous when effectors are constitutively produced (plasmid fusions); (ii) under more physiological conditions (chromosomal fusions) SlrP can be detected at short and long times p.i., while SspH1 and SspH2 tend to be secreted only at long times; (iii) translocation is T3SS2-dependent for RAW264.7 at 8 h.

### 3.4 Role in host cell invasion and intracellular proliferation

**Figure 4:**
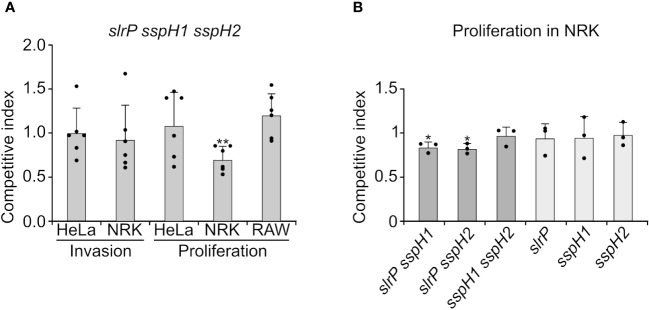
Effect of SlrP, SspH1, and SspH2 on host cell invasion and proliferation. **(A)** Analysis of invasion and intracellular proliferation of the triple mutant *slrP sspH1 sspH2* in mixed infections with a *trg*::Mu*d*J mutant used as the wild-type strain. **(B)** Analysis of intracellular proliferation in NRK cells of the indicated mutants in mixed infections with a *trg*::Mu*d*J mutant used as the control strain. Competitive indices are the means of three infections. The error bars represent the standard deviations. Dots represent individual values. Asterisks denote that the indices are significantly different from 1 for a t-test: *P-value < 0.05, **P-value < 0.01.

To test the relevance of effectors of the NEL family in cell invasion and intracellular proliferation, competitive indices were calculated after mixed infections of host cells with the triple mutant *slrP sspH1 sspH2* and a strain carrying a *trg::lacZ* fusion. The *trg* mutant has no invasion or proliferation defect (29) and forms blue colonies in X-Gal supplemented plates, providing a way to distinguish between colonies of both strains. A ratio 10:1, *slrP sspH1 sspH2*: *trg::lacZ*, was used for the infection input in order to prevent the possibility of complementation of the triple mutant by the control strain. Invasion and proliferation were tested in epithelial HeLa cells and NRK fibroblasts. Proliferation was also studied in RAW264.7 macrophages. As seen in **Figure 4A**, a significant defect was only detected for proliferation inside NRK cells. Then we measured intracellular proliferation in NRK of single and double mutants. Although single mutants were not defective, double mutants *slrP sspH1* and *slrP sspH2* exhibited a slightly but significantly reduced ability to proliferate inside these cells (**Figure 4B**). These results suggest the existence of a certain redundancy between the three effectors of this family and an additive effect of the mutations studied for this particular phenotype.

### 3.5 Specificity of interactions and ubiquitination of known substrates

**Figure 5:**
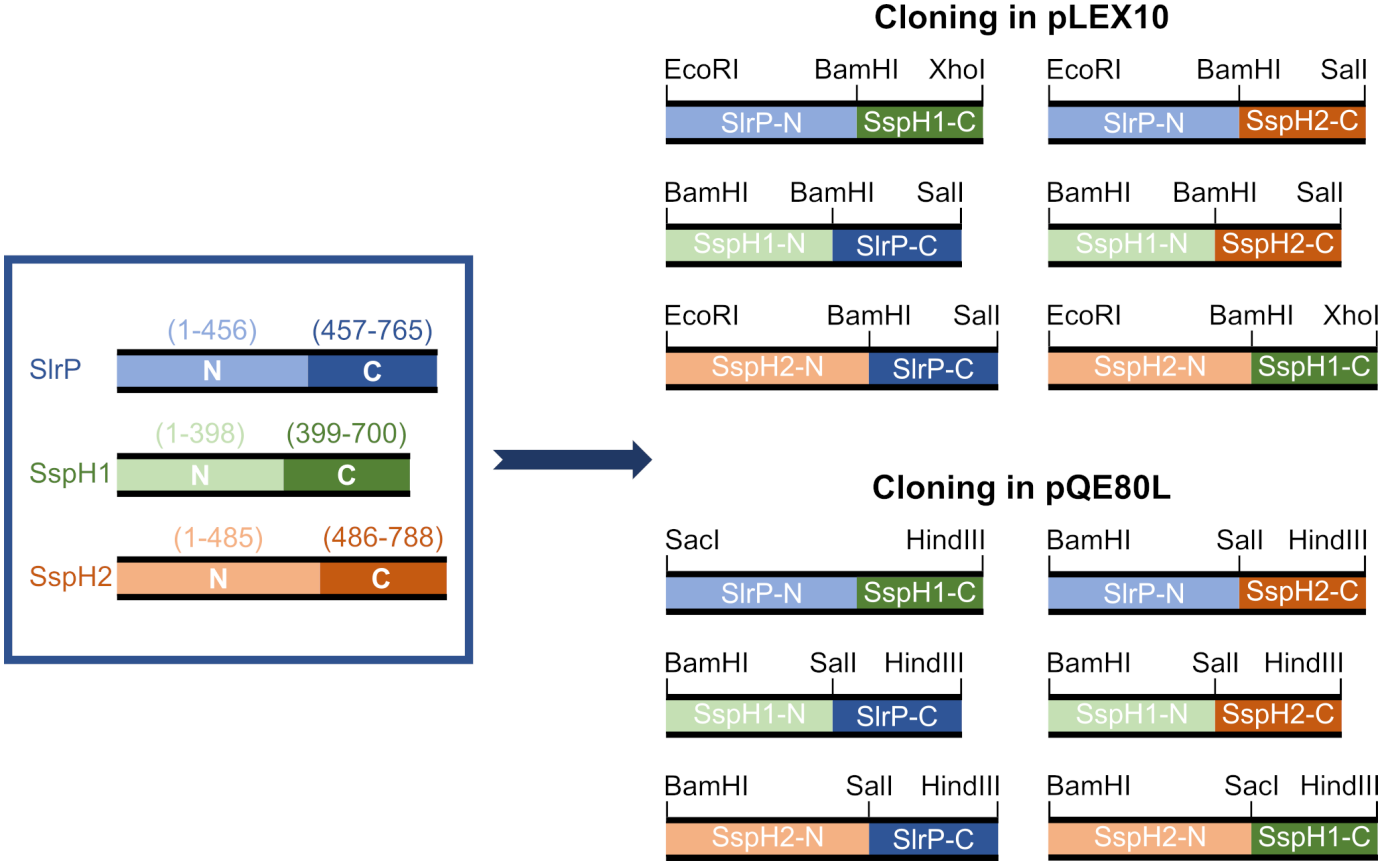
Design of clones for the expression of chimeric NEL effectors. The represented N- and C-terminal fragments of SlrP, SspH1 and SspH2 were cloned into the vectors pLEX10 (for yeast two-hybrid experiments) and pQE80L (for 6His fusions generation) using the indicated restriction enzymes.

Effectors of the NEL family are typically composed of a N-terminal secretion motif, a leucine-rich repeat (LRR) domain, and a C-terminal novel E3 ubiquitin ligase (NEL) domain (16). While the NEL domain is necessary for the ubiquitination of host proteins, the LRR domain is supposed to be involved in the interaction with substrates. We wondered whether the selection of substrates relied entirely on this domain. To test this hypothesis, we cloned DNA fragments encoding the N- or C-part of SlrP, SspH1 and SspH2, and combinations of these fragments to generate all possible chimeric proteins (**Figure 5**). We used the vector pLEX10 to test interactions using the yeast two-hybrid system and the vector pQE80L to produce and purify proteins with the 6-His tag and test them in ubiquitination assays.

**Figure 6:**
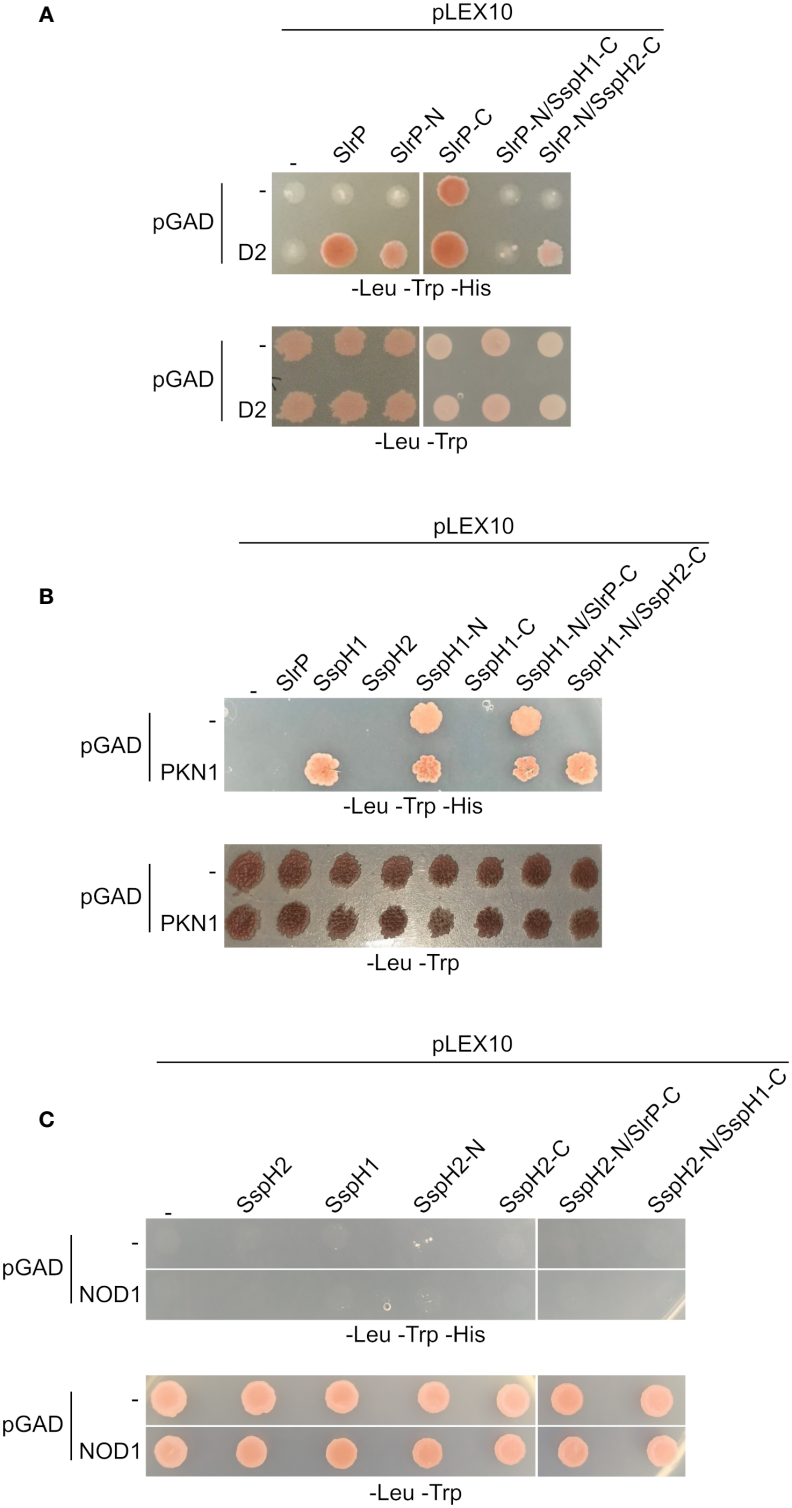
Interaction of SlrP, SspH1 and SspH2 with host proteins in the yeast two-hybrid system. Derivatives of pLEX10 and pGAD1318 (pGAD) were introduced into yeast strain L40 by transformation. Transformants were selected in media lacking tryptophan and leucine. **(A)** Study of the interaction of SlrP or derivatives in which the C-terminal part was replaced by that of SspH1 or SspH2 with SNRPD2 (D2). **(B)** Study of the interaction of SspH1 or derivatives in which the C-terminal part was replaced by that of SlrP or SspH2 with PKN1. **(C)** Study of the interaction of SspH2 or derivatives in which the C-terminal part was replaced by that of SlrP or SspH1 with NOD1.

Physical interactions were studied for cognate substrates of these effectors: SNRPD2 (21) for SlrP, PKN1^HR1b^ subdomain (44, 45) for SspH1 and NOD1 (24) for SspH2. The C-terminal fragment of SlrP (SlrP-C) and the N-terminal fragment of SspH1 (SspH1-N) exhibited autoactivation problems that precluded the analysis of interactions in the yeast two-hybrid system (**Figure 6**). However, some interesting conclusions can be drawn from this analysis. (i) The N-terminal fragments (that included the LRR domains) of SlrP and SspH1 direct the interaction with SNRPD2 and PKN1, respectively, alone or in fusion with the C-terminal domain of SspH2. (ii) No interaction was detected for NOD1, which may be due to the necessity of the formation of a ternary complex between SspH2, NOD1 and SGT1 (24).

**Figure 7:**
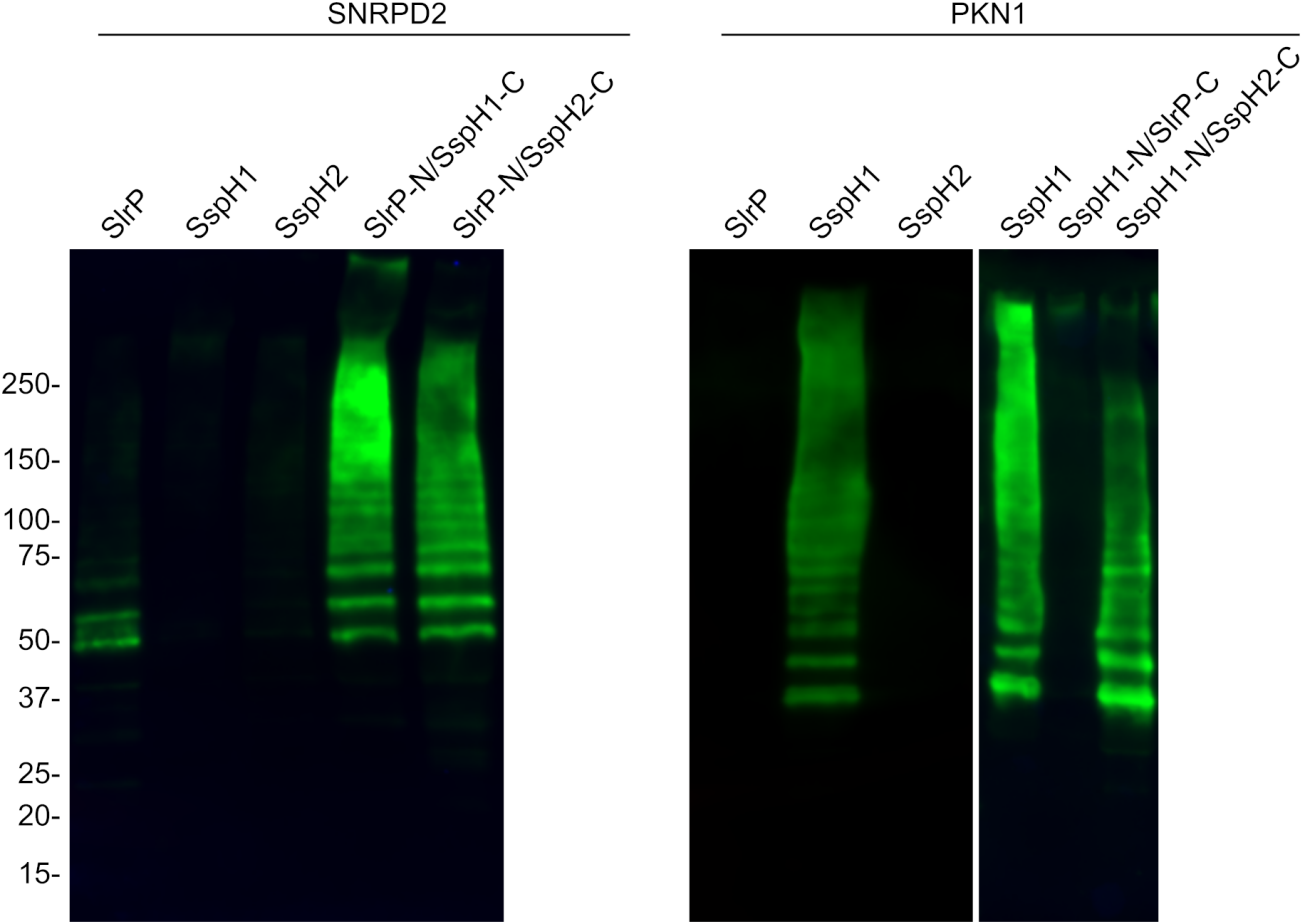
Ubiquitination of SNRPD2 and PKN1 by SlrP, SspH1, SspH2 and chimeric effectors. The ubiquitination of GST-SNRPD2 or GST-PKN1 bound to glutathione-agarose beads was tested in the presence of HA-ubiquitin, E1, E2, and a *Salmonella* effector fused to 6His. The beads were washed prior to immunoblot analysis. The sizes in kDa of the molecular weight markers are shown on the left. Replicate experiments for this figure are shown in **Supplementary Material**.

In view of these results, we decided to focus on the study of the ubiquitination of SNRPD2 and PKN1. *In vitro* ubiquitination assays shown in **Figure 7** indicated that SNRPD2 can be ubiquitinated by SlrP, but not by SspH1 or SspH2, as previously described (21). Interestingly, this host protein is also ubiquitinated in the presence of the chimeric proteins SlrP-N/SspH1-C and SlrP-N/SspH2-C. In addition, PKN1 can be ubiquitinated by SspH1, but not SlrP or SspH2, and by the chimeric protein SspH1-N/SspH2-C. These results are fully consistent with the conclusions obtained from the physical interaction analysis and support our hypothesis that substrate specificity for these effectors resides in the LRR domain.

**Figure 8:**
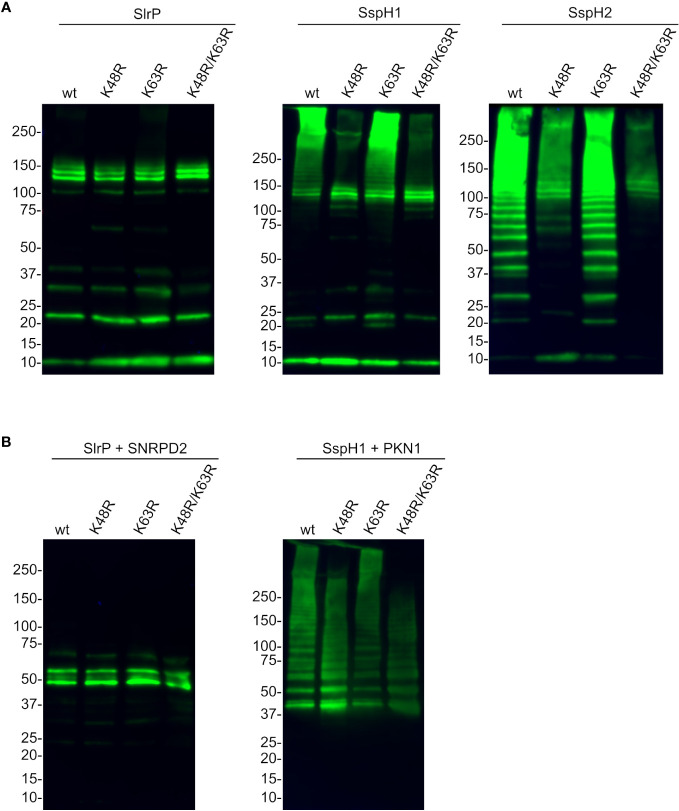
Analysis of the specific type of polyubiquitination catalyzed by SlrP, SspH1, and SspH2. HA-ubiquitin and its derivatives with the indicated mutations replacing lysines by arginines (K48R, K63R, K48R/K63R) were used in ubiquitination assays with 6His fusions of SlrP, SspH1, and SspH2. **(A)** No additional substrate was added and the whole reaction was analyzed to detect polyubiquitination of ubiquitin. **(B)** GST-SNRPD2 or GST-PKN1 bound to glutathione-agarose beads were added as substrates and beads were washed prior to immunoblot analysis with anti-HA antibodies. The sizes in kDa of the molecular weight markers are shown on the left. Replicate experiments for this figure are shown in **Supplementary Material**.

Lysines 48 and 63 are the main ubiquitin residues involved in the formation of polyubiquitin chains and the use of a particular Lys results in different outcomes in terms of stability or activity of the ubiquitinated protein. Therefore, we next produced different forms of HA-tagged ubiquitin: wild-type ubiquitin and mutants with specific Lys changed into Arg (K48R, K63R and K48R/K63R). Then we used these ubiquitin forms in ubiquitination assays with the three effectors (**Figure 8**). The results of these experiments suggest that, while the polyubiquitination catalyzed by SspH1 and SspH2 is at least partially dependent on Lys 48, other lysines may be used by SlrP.

### 3.6 Effects in cytokine secretion

**Figure 9:**
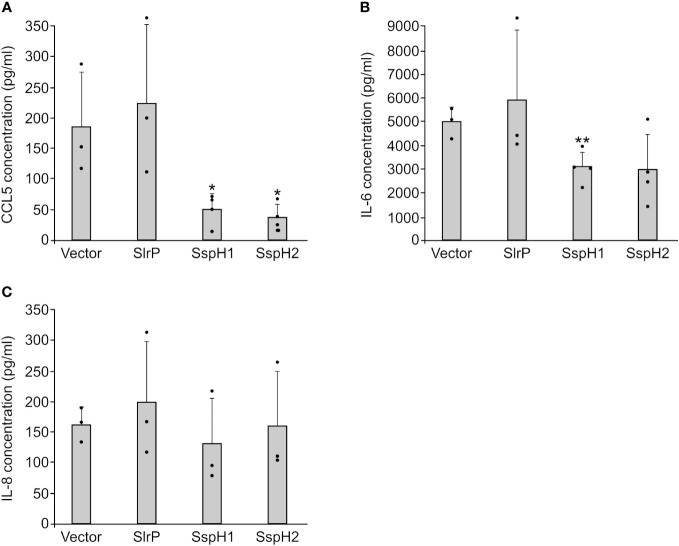
Effect of SlrP, SspH1 and SspH2 on the secretion of cytokines by mammalian cells. HeLa cells were transfected with a plasmid expressing SlrP, SspH1 or SspH2 or with the empty vector and the concentration of CCL5 **(A)**, IL-6 **(B)**, and IL-8 **(C)** was measured in the supernatants of cell cultures 24 h after transfection. Data are presented as mean values + standard deviations of at least three biological replicates. Dots represent individual values. * *p* < 0.05, ** *p* < 0.01, for a two-tailed Student’s *t*-test comparing cells expressing each effector with cells transfected with the empty vector.

Previous studies have shown that SspH1 inhibits IL-8 production in host cells (46). In contrast, SspH2 enhances IL-8 secretion (24) and may decrease expression of genes that encode several cytokines (47). These experiments were carried out using different methodologies and different host cell models. Therefore, we decided to measure the effect of SlrP, SspH1, and SspH2 on the secretion of several cytokines using a comparable approach. Human HeLa cells were transfected with plasmids expressing *slrP*, *sspH1*, *sspH2*, or the empty vector as a control. Then, CCL5, IFN-γ, IL-1β, IL-6 and IL-8 concentrations were measured in the supernatants of cell cultures 24 h after transfection. Secretion of IFN-γ and IL-1β was not detected in this model. As seen in **Figure 9**, while SlrP had no effect on the secretion of the assayed cytokines, SspH1 and SspH2 caused a decrease in the secretion of CCL5 and IL-6, suggesting an anti-inflammatory role for these effectors.

## 4 Discussion

*Salmonella* injects more than 40 effectors into host cells to manipulate their behavior and facilitate the survival and replication of the pathogen. Some of these effectors act as adaptor proteins or contribute to modifying the lipid content of host membranes, but most of these effectors exhibit some biochemical activity that mediates post-translational modifications of host proteins (48). These modifications include ubiquitination, deubiquitination, phosphorylation, dephosphorylation, hydrolysis, deamidation, glycosylation, and acetylation. Interestingly, there are effectors that share biochemical activity and have similarities in sequence and structure, so that they are part of the same family. Examples of effector families found in *S. enterica* serovar Typhimurium are the family of effectors with arginine N-glycosyltransferase activity, with SseK1, SseK2 and SseK3 (49), and the NEL family of E3 ubiquitin ligases, with SlrP, SspH1 and SspH2 (16). Redundancies can provide an additional level of robustness to the *Salmonella* virulence strategy: if one effector is inactivated by host defenses, another one can carry out the function. However, there are several ways in which members of the same family can accomplish different missions: (i) they can differ in the promoter region of the genes encoding them, and so their regulation may be different; (ii) the N-terminal portion of the protein, which directs translocation, may be adapted to a particular T3SS apparatus, or the timing of expression of the protein can restrict translocation to that particular apparatus; (iii) specific domains can direct selection of interaction and biochemical targets; (iv) subcellular localization can be different and may lead to functional specialization.

This study was initiated to analyze all these aspects in the case of the NEL family of E3 ubiquitin ligases. Regarding regulation, our experiments show that synthesis of the three effectors is totally dependent on PhoP *in vitro* under SPI2-inducing conditions (**Figure 1**). PhoP is the cytosolic response regulator of the two-component regulatory system PhoQ/PhoP. The membrane-associated sensor PhoQ is activated by a variety of signals, including low periplasmic Mg^2+^ concentration, mildly acidic pH in the cytosol, increased osmolarity, and sublethal concentrations of cationic antimicrobial peptides (50). Once activated, PhoP is a master regulator of *Salmonella* virulence. It represses the transcription of *hilA*, which encodes the major transcriptional activator of SPI1 genes, and activates the transcription of *ssrB*, whose product is the response regulator of the SsrA/SsrB two-component regulatory system, which is necessary for the expression of SPI2 genes. Interestingly, SsrB is essential for the transcription of *sspH2*, whereas the regulation of *slrP* and *sspH1* by SsrB is only partial (**Figure 1B**). The results obtained with transcriptional *lux* fusions were confirmed at the protein level by immunoblotting (**Figure 1C**). The expression of the genes encoding the three effectors is also dependent on PhoP *in vivo* during macrophage infection (**Figure 2**). Interestingly, transcription of *sspH2* is PhoP independent inside HeLa and NRK cells. These results suggest that there may be other signals inside these cells that are able to activate SPI2 expression. This is consistent with previous data showing, for instance, that the PhoQ/PhoP pathway is not required for SPI2 expression in the presence of phosphorylated OmpR (51).

The expression and regulation pattern of *sspH2* is consistent with its translocation into host cells at 8 h p.i., when SPI2 is expressed, but not 2 h p.i., when only SPI1 has been expressed during invasion (**Figure 3B, D, F**). In contrast, SlrP translocation was detected at 2 h and 8 h p.i. since, as previously described (18), it is expected to be translocated both by T3SS1 and T3SS2. The timing of translocation of SspH1 is more similar to that observed for SspH2 and appears to occur preferentially through T3SS2. In these experiments, we expressed the genes encoding the effectors from their native promoters (CyaA’ fusion in the chromosome) or from a constitutive promoter (CyaA’ fusions in a plasmid). This analysis provides an additional conclusion: the three effectors have the ability to be translocated by T3SS1 and T3SS2, only the lack of expression under native conditions prevents SspH1 and SspH2 from being translocated through T3SS1 (compare, for instance, **Figures 3A, B** at 2 h p.i.). Translocation was studied in three different cell lines: HeLa (human epithelial), RAW264.7 (murine macrophages), and NRK (rat fibroblasts). The diverse patterns observed can be attributed, at least in part, to the different ways of entry used by *Salmonella*. Invasion of HeLa cells occurs only by the trigger system induced by T3SS1. Therefore, a *prgH* mutant, lacking T3SS1, is unable to invade and so translocation of effectors cannot be detected at any time in these cells (**Figure 3B**). In contrast, the *prgH* mutant is capable of entering macrophages by phagocytosis and can translocate SlrP, SspH1, and SspH2 into these cells at 8 h p.i., but translocation is lost in the *ssaV* mutant, indicating that T3SS2 is the relevant system under these conditions (**Figure 3D**). The situation is more complex in fibroblasts, with significant secretion of SlrP through both systems (**Figure 3F**, the double mutation is needed to prevent translocation). This can be explained because *Salmonella* invades fibroblasts through multiple routes (52). It should be taken into account that the method used to detect translocation of effectors into host cells has some limitations. In particular, in HeLa and NRK cells the interpretation is more difficult than in RAW264.7 cells because we cannot clearly separate T3SS1-dependent from T3SS2-dependent secretion since T3SS1 expression (invasive conditions) are needed in order to infect these cells. Previous data indicated that half-lives of effectors of this family are longer than several hours (53, 54). As a consequence, the high levels of translocation observed for SlrP in HeLa cells (**Figure 3B**) at 8 h with the *ssaV* mutant (lacking T3SS2) could be due to the translocation that occurred previously through T3SS1.

The multifactorial nature of bacterial virulence makes it prone to be affected by redundancy phenomena. In fact, the lack of only one of the more than 40 T3SS effectors, which can be obtained with a single null mutant, usually does not lead to a significant decrease in *Salmonella* pathogenicity. This is what we observe for SlrP, SspH1, and SspH2 in our *in vitro* cellular infection models (**Figure 4**). However, a mutant lacking the three effectors is significantly attenuated for intracellular proliferation in NRK cells compared to wild-type bacteria. These results suggest that these three effectors work redundantly to allow *Salmonella* survival inside fibroblasts. They also show that there are different requirements and mechanisms for the adaptation of this pathogen to different host cell lines since no defects were detected in epithelial cells or macrophages.

SlrP, SspH1, and SspH2 share a similar structure and a similar autoinhibition mechanism in which the activity of the NEL domain is repressed by the LRR domain in the absence of substrate (55). There are several well defined host substrates for these effectors that include SNRPD2, PKN1, and NOD1, respectively. We have studied the level of specificity in the interaction and ubiquitination of these substrates and the determinants of this specificity. To do that, we have generated all possible combinations of the LRR and NEL domains of the three effectors (**Figure 5**) and tested their interaction with host proteins using the yeast two-hybrid system. Our previous results showed that SNRPD2 interacts specifically with SlrP but not with SspH1 or SspH2 (21). Here, we show that the N-terminal region of SlrP, including the LRR domain, directs this interaction (**Figure 6A**). Analogous results were obtained for PKN1, whose Hr1b domain interacts specifically with SspH1. In this case, the interaction with the isolated N-terminal region, containing the LRR domain, could not be tested due to transactivation of the reporter gene in the yeast two-hybrid system. However, the fact that this interaction occurs through this part of the protein was confirmed by the chimeric protein SspH1-N/SspH2-C, which did not show transactivation (**Figure 6B**). These results confirm the notion that host proteins interact with these effectors through their LRR domains. More interestingly, the chimeric proteins SlrP-N/SspH1-C and SlrP-N/SspH2-C were able to ubiquitinate SNRPD2 (**Figure 7**), indicating that the NEL domains of the three effectors can catalyze the ubiquitination of this host target if fused to the interaction determinant provided by the LRR domain of SlrP. In fact, the NEL domains of SspH1 and SspH2 demonstrated greater ubiquitination activity on SNRPD2 than the NEL domain of SlrP under these conditions. Similar results were obtained for PKN1 and SspH1, although in this case ubiquitination was not detected with the chimeric protein SspH1-N/SlrP-C. This may be due to lower activity of the catalytic domain of SlrP or to an improper folding of the chimera.

Our ubiquitination experiments showed in all cases the classical ladders that are indicative of polyubiquitination. The type of linkage between ubiquitins within the ubiquitin polymer determines the fate of the marked protein. As mentioned above, K48-linked chains are typically related to proteasomal degradation, while K63-linked chains are generally involved in signaling processes. In order to find out the type of polyubiquitination that was catalyzed by each effector, we generated and used mutant forms of ubiquitin. Assays carried out without a substrate, where polyubiquitination of ubiquitin is observed, revealed a strong dependence on Lys48 for the activity of SspH2 and also SspH1, although in the latter the effect is observed more clearly in very high molecular weight polyubiquitin bands (**Figure 8A**). The pattern of SlrP-induced polyubiquitination did not change dramatically using K48R or K63R mutant ubiquitins, suggesting that other residues may be involved. In fact, ubiquitin has five additional lysine residues (K6, K11, K27, K29, and K33) that, together with methionine 1 (M1), can be ubiquitinated. Ubiquitination assays using SNRPD2 or PKN1^HR1b^ as substrates confirm these conclusions (**Figure 8B**).

*S. enterica* serovar Typhimurium manipulates host inflammatory responses in complex ways (56, 57). Stimulation of inflammatory signaling depends on the T3SS effectors SopB, SopE, and SopE2, which act at the beginning of infection to help *Salmonella* compete with the normal intestinal microbiota. However, to prevent an excessive inflammatory response that would be detrimental to the bacteria, *Salmonella* limits this response by expressing other effectors, including SptP, PipA, GogA, GtgA and AvrA (58–61). SspH1 from *S. enterica* serovar Typhimurium also down-regulates the production of the pro-inflammatory factor IL-8 (46). In contrast, SspH2 was shown to enhance IL-8 secretion (24), and the effect of SlrP was not previously reported. Here, we studied the effect of the three effectors on several pro-inflammatory cytokines. Although we did not detect significant effects on IL-8 secretion, there was a reduction in CCL5 and IL-6 secretion in the presence of SspH1 or SspH2, but not SlrP (**Figure 9**). Previous experiments in a mouse model of anorexia suggested that SlrP from *S. enterica* serovar Typhimurium inhibited inflammasome activation and IL-1β maturation in the small intestine (62). Furthermore, SspH2 from *S. enterica* serovar Enteritidis was shown to reduce the expression of pro-inflammatory factors, including IL-1β, IFN-γ, and iNOS, in Caco-2 BBE cells (47). Together, these results and the results presented here support a predominant anti-inflammatory role for *Salmonella* NEL effectors that can be expressed in different ways depending on the biological model and context. Interestingly, SlrP and SspH2 are involved in the inhibition of antigen presentation in dendritic cells (63) and in preventing the migration of these cells toward the chemokine CCL19 (64). Furthermore, *Salmonella* has been shown to interfere with MHC class II antigen presentation, inducing polyubiquitination and reduction of cell surface HLA-DR expression (65). This effect depends on T3SS2, so it is tempting to speculate with the involvement of *Salmonella* NEL effectors. However, previous experiments failed to identify them as potential candidates and showed that the T3SS2 effector SteD decreased surface levels of MHC class II in dendritic cells by recruiting the host E3 ubiquitin ligase Wwp2 (66, 67). Further experiments will be required to clarify the role of these effectors in this function.

In conclusion, despite their similar structure and biochemical activity, the three members of the NEL family of T3SS effectors of *S. enterica* serovar Typhimurium have different expression and translocation patterns and recognize specific host targets that are modified with different polyubiquitination patterns. Unfortunately, few targets of these effectors have been described so far. Therefore, additional research is needed to obtain a complete list of these targets and a clearer picture of all the effects of these *Salmonella* proteins on the host.

## Data availability statement

The original contributions presented in the study are included in the article/**Supplementary Material**. Further inquiries can be directed to the corresponding authors.

## Author contributions

AB-B: Formal analysis, Investigation, Methodology, Visualization, Writing – review & editing. PM-M: Formal analysis, Investigation, Methodology, Visualization, Writing – review & editing. CV-G: Formal analysis, Investigation, Methodology, Visualization, Writing – review & editing. JB-B: Formal analysis, Funding acquisition, Project administration, Resources, Supervision, Writing – review & editing. FR-M: Formal analysis, Funding acquisition, Resources, Supervision, Writing – original draft, Writing – review & editing.
