# Supplementary material for: Specificities and redundancies in the NEL family of bacterial E3 ubiquitin ligases of Salmonella enterica serovar Typhimurium
Source: Front Immunol. 2024 Feb 1;15:1328707. doi: 10.3389/fimmu.2024.1328707 (PMC10867120; doi:10.3389/fimmu.2024.1328707)
Supplement: Supplementary file 1 [file DataSheet_1.pdf]

Supplementary Material

Specificities and redundancies in the NEL family of bacterial E3 ubiquitin ligases of *Salmonella enterica* serovar Typhimurium

Andrea Bullones-Bolaños[†], Paula Martín-Muñoz[†], Claudia Vallejo-Grijalba[†], Joaquín Bernal-Bayard*, Francisco Ramos-Morales*

*** Correspondence:** Joaquín Bernal-Bayard, jbbayard@us.es; Francisco Ramos-Morales, framos@us.es

Figure 1C-1

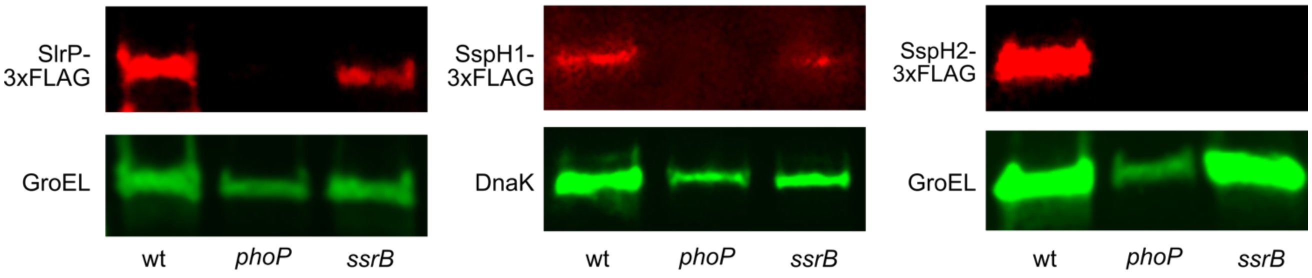

Figure 1C-2

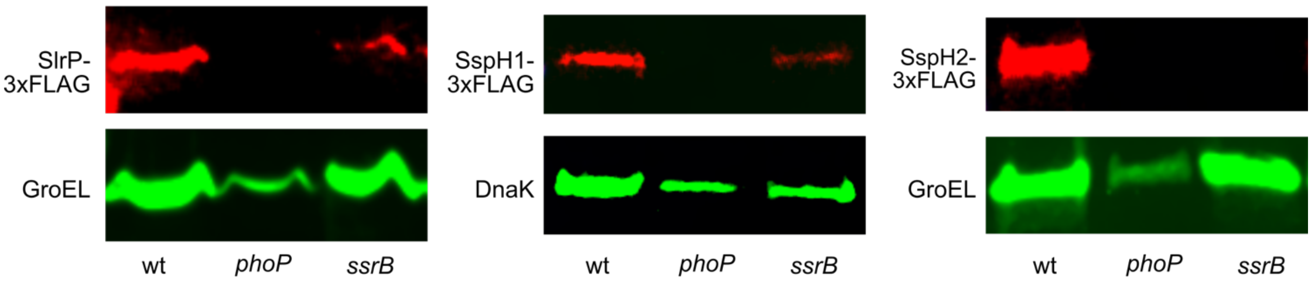

Figure 1C-3

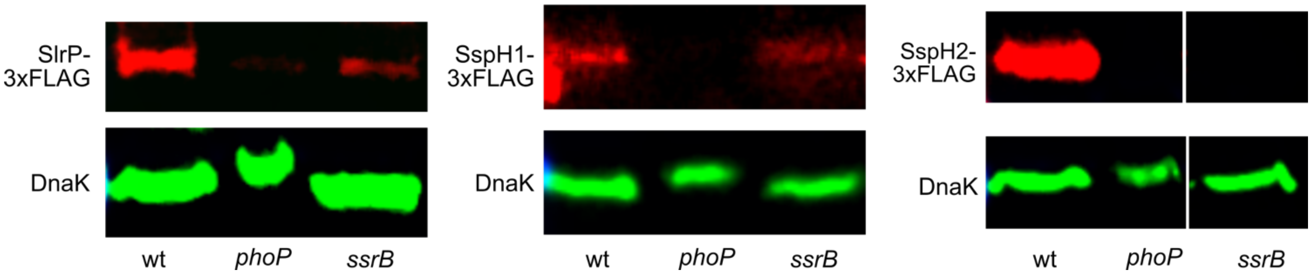

Supplementary Figure 1C. Replicate experiments for Figure 1C. Expression and regulation of *slrP*, *sspH1*, and *sspH2*. (C) Protein extracts of derivatives of *S. enterica* serovar Typhimurium strains expressing 3xFLAG-tagged SlrP, SspH1, or SspH2 grown under SPI2-inducing conditions were resolved by SDS-PAGE. Immunoblotting was performed with monoclonal anti-FLAG antibodies. Anti-GroEL or anti-DNAK antibodies were used as loading control.

Figure 7-1

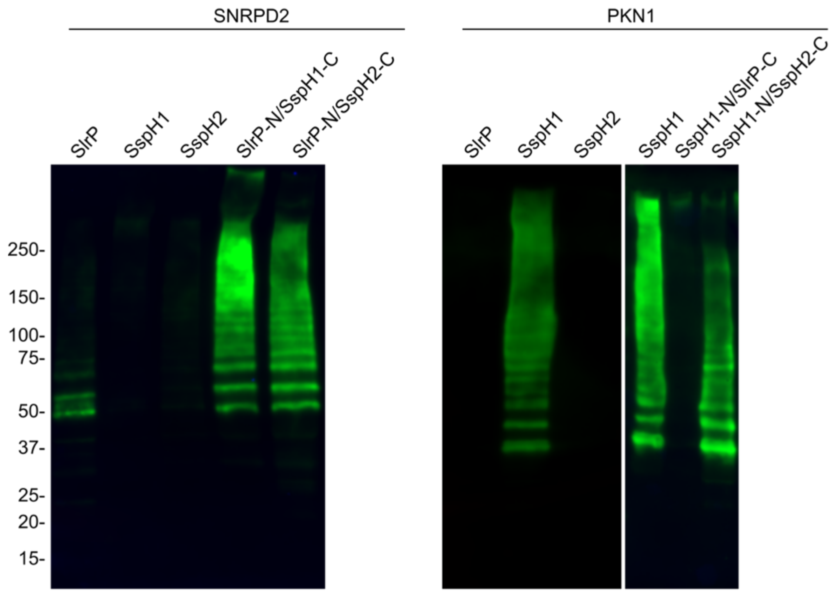

Figure 7-2

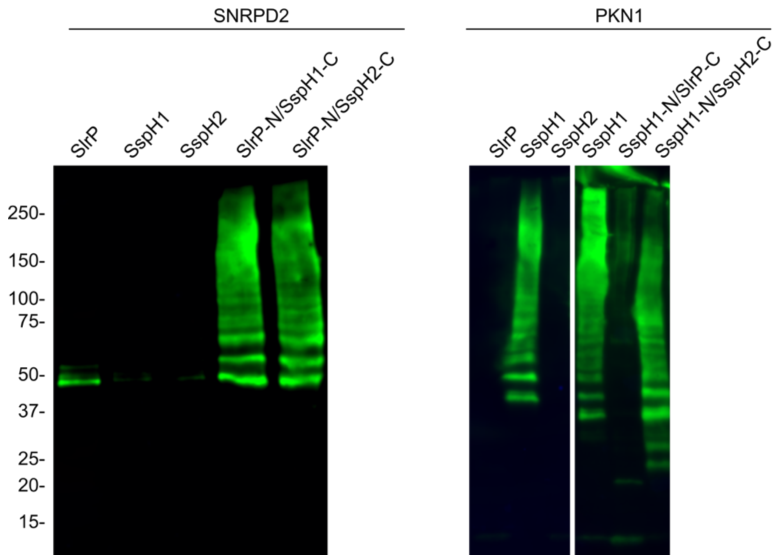

Supplementary Figure 7. Replicate experiments for Figure 7. Ubiquitination of SNRPD2 and PKN1 by SlrP, SspH1, SspH2 and chimeric effectors. The ubiquitination of GST-SNRPD2 or GST-PKN1 bound to glutathione-agarose beads was tested in the presence of HA-ubiquitin, E1, E2, and a *Salmonella* effector fused to 6His. The beads were washed prior to immunoblot analysis. The sizes in kDa of the molecular weight markers are shown on the left.

Figure 8A-1

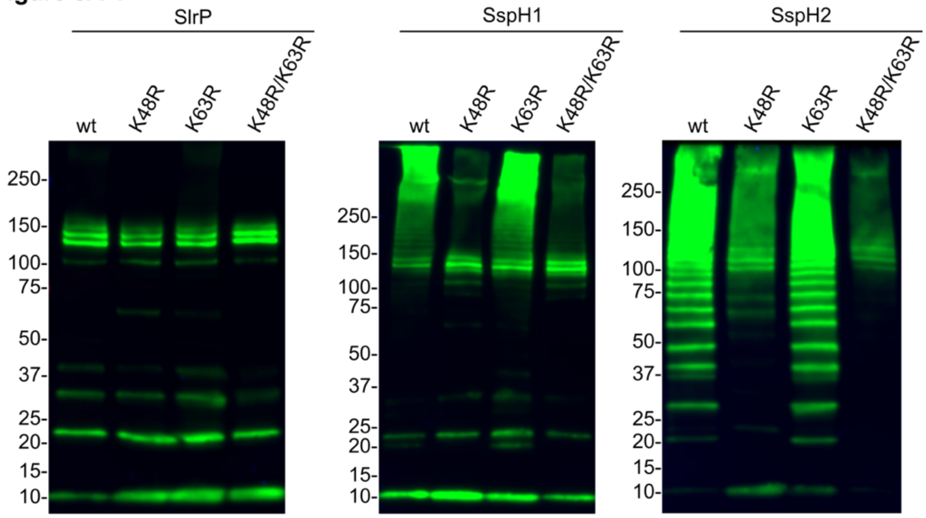

Figure 8A-2

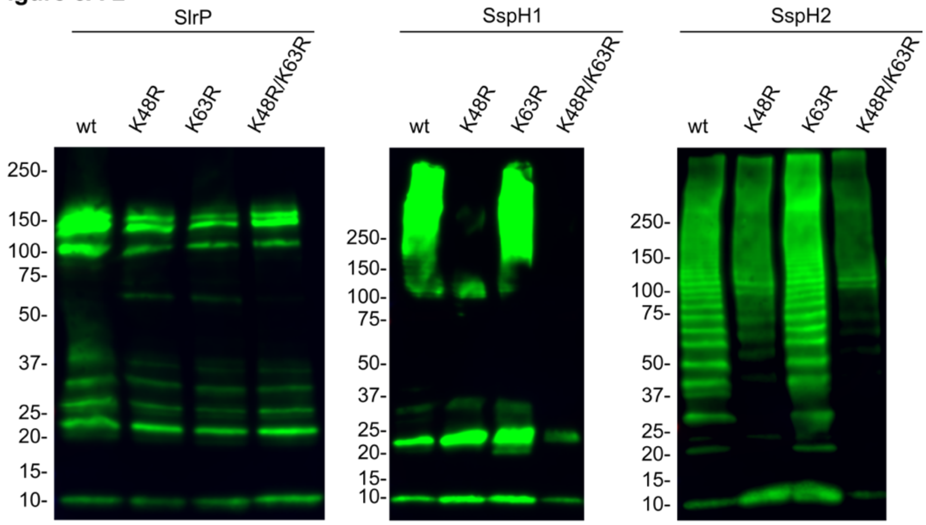

Figure 8A-3

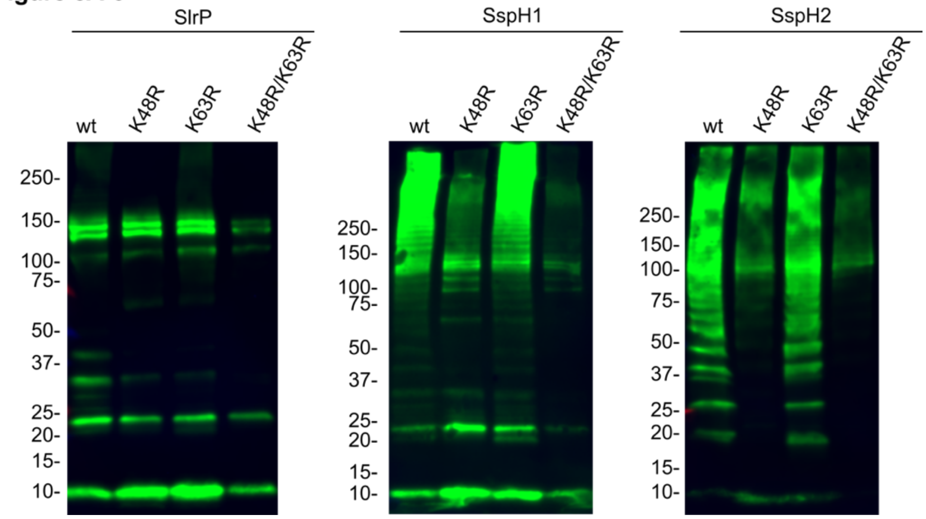

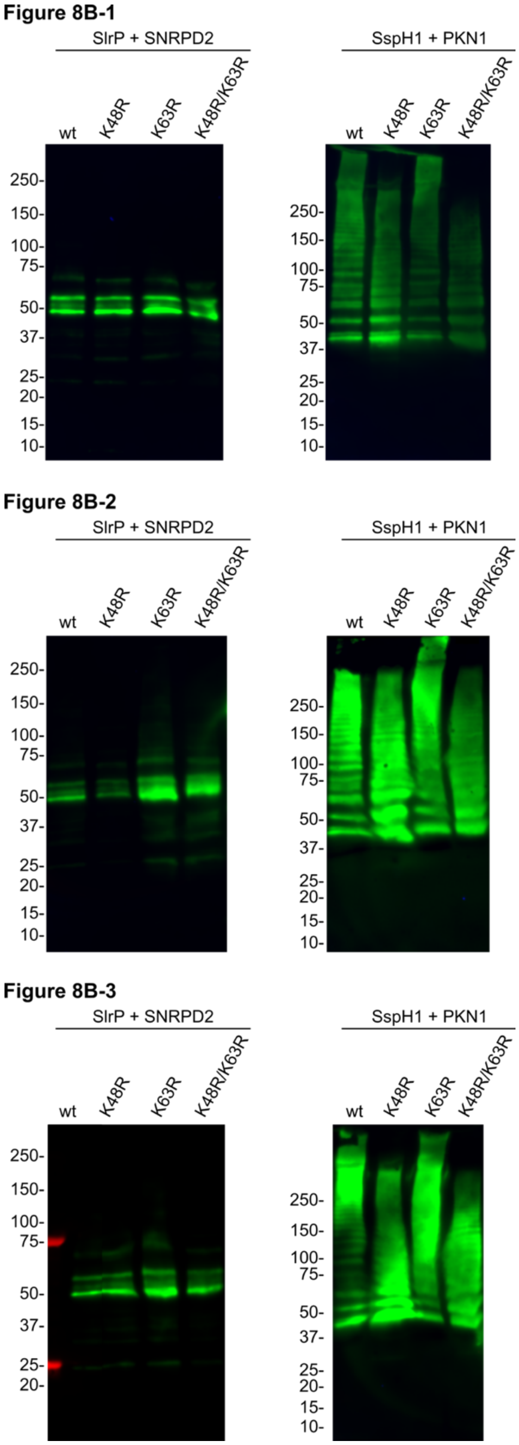

Supplementary Figure 8. Replicate experiments for Figure 8. Analysis of the specific type of polyubiquitination catalyzed by SlrP, SspH1, and SspH2. HA-ubiquitin and its derivatives with the indicated mutations replacing lysines by arginines (K48R, K63R, K48R K63R) were used in ubiquitination assays with 6His fusions of SlrP, SspH1, and SspH2. (**A**) No additional substrate was added and the whole reaction was analyzed to detect polyubiquitination of ubiquitin. (**B**) GST-SNRPD2 or GST-PKN1 bound to glutathione-agarose beads were added as substrates and beads were washed prior to immunoblot analysis with anti-HA antibodies. The sizes in kDa of the molecular weight markers are shown on the left.
